# A journal for the modern era

**DOI:** 10.1002/edm2.1

**Published:** 2017-07-04

**Authors:** Mark Gurnell

We live in a time of “now,” when we can pretty much find anything we need at the click of a button, have it delivered to our door within hours, and often without the need to compromise on quality. The market place is more competitive than ever – as a supplier, if you can't deliver a quality product in a timely manner, then somebody else will step into the gap and, before you know it, the opportunity has closed.

Just like every other aspect of modern life, endocrinology, diabetes and metabolism are fast moving with new, often ground‐breaking, developments emerging every week, if not every day. Technological advances mean that many basic scientific and clinical questions can now be addressed in a way that could only have been dreamt about as recently as five years ago. However, identifying which are the important questions to ask, and working out how best to address these, remains as big a challenge as ever.

So, when we complete a piece of work, irrespective of whether the findings are “positive” or “negative,” we quite rightly want to get our message out and into the public domain – but then the next challenge appears – how best to do this? For most of us, publishing in scientific journals remains important – yes, there are alternative ways to “publish” our findings, especially if we are sufficiently adept to exploit the opportunities presented by social media – but, at the end of the day, the traditional model, whereby our work is subjected to pre‐publication scrutiny by our peers (who can hopefully point out the highlights, as well as the potential shortcomings!), remains as important as ever. Indeed, good peer review often adds to the quality of the final product – who hasn't been grateful for the insightful comments of a reviewer or editor that have improved the quality of an article, and for which we can take the credit!

However, publishing in scientific journals remains as competitive as ever, with researchers aspiring to see their labours rewarded with acceptance by one of the “higher ranked” journals. Of course, such an approach is not without its challenges. The acceptance rates in many journals remain relatively low – and often this isn't simply a reflection of the quality of the material submitted, but rather a “lack of space” such that many worthy articles are turned away, especially if they are not deemed sufficiently novel or are viewed as simply confirming the work of others. However, this strategy is flawed because good quality confirmatory papers, or indeed studies that refute the findings of previous manuscripts, may be just as important as the original work. Indeed, the topic of reproducibility of medical scientific discovery is currently a “live debate” as public and other funding bodies seek to ensure they are securing full value for money from their investments.

Another issue facing the modern researcher is how to navigate the challenge of Open Access – again many funding bodies and host institutions demand that their grant holders/employees make their work available as soon as possible using the Open Access model.

So, when approached by Wiley to consider the introduction of a new journal, into what already seems a congested market place, the most important question to ask was whether the model proposed would create a journal that could meet the demands of publishing in the modern era. I'm pleased to say that Endocrinology, Diabetes & Metabolism has been designed to be just that. Like other journals, ultimately the quality of material submitted will be the key factor in deciding whether an article is accepted for publication – so, for a primary data manuscript the validity of the hypothesis/aims, methods, results and discussion will all remain central to journal decision making, and similarly for reviews and other articles, quality will be the key watchword. However, while novelty will still remain important, it will not be an essential requirement for publication in the journal.

Endocrinology, Diabetes & Metabolism will accept manuscripts from across the full breadth of our specialty, ranging from basic science to clinical practice, and in so doing will hopefully offer something for everyone.

As an Open Access journal one of our priorities will be to ensure timely publication in a manner that allows authors to readily meet their obligations to funding bodies and host institutions.

Finally, although the on‐line environment offers the option to “do away” with word and figure limits, the Editorial Team believes strongly that the ability to distil a message into a compact format remains an essential skill and one that benefits all – in the modern world everyone is busy, our attention spans limited, and so delivering our message rapidly, in a readily digestible format, is likely to pay significant dividends all round.

